# Investigating Preventive Behaviors Toward COVID-19 Among Iranian People

**DOI:** 10.3389/fpubh.2021.590105

**Published:** 2021-02-16

**Authors:** Fatemeh Baghernezhad Hesary, Hamid Salehiniya, Mohammadreza Miri, Mitra Moodi

**Affiliations:** ^1^Social Determinants of Health Research Center, Birjand University of Medical Sciences, Birjand, Iran; ^2^Department of Epidemiology and Biostatistics, Social Determinants of Health Research Center, Birjand University of Medical Sciences, Birjand, Iran; ^3^Social Determinants of Health Research Center, Department of Health Education and Health Promotion, School of Health, Birjand University of Medical Sciences, Birjand, Iran

**Keywords:** COVID-19, health behavior, prevention, education, physical activity

## Abstract

**Background:** Considering the importance of preventive behaviors in reducing the transmission of COVID-19, this study was conducted to determine the preventive behaviors toward the spread of COVID-19.

**Methods:** This cross-sectional study was performed 7 weeks after the confirmation of the first case of Covid-19 by the Ministry of Health and Medical Education in Iran. Data were completed online using a researcher-made questionnaire, the validity and reliability of which were confirmed, for 1,200 Iranians from 8 April 2020 to 9 May 2020.

**Results:** The mean age of study participants was 37.77 ± 11.20 years. The mean score of preventive behaviors was 62.67 ± 8.53. The results showed that there was a significant relationship between the variables of gender, education, economic status, and preventive behaviors of COVID-19. The highest frequency is related to not using hookah and cigarettes and then avoiding losing and rubbing (78%) and the lowest percentage is related to exercising behavior (16%).

**Conclusion:** Due to the low level of prevention behaviors during the outbreak of the disease, it is important to design educational and supportive interventions to improve and perform coronavirus prevention behaviors with more focus on men, illiterate people, and poor economic status.

## Background

Coronaviruses are a large family of RNA viruses that cause disease in humans and animals. These viruses can cause a wide range of respiratory illnesses such as SARS, MERS, and common cold (1). The COVID-19 epidemic is a newly emerging infectious disease virus that was identified in Wuhan China in late 2019 and then spread rapidly across the world (2). Coronavirus is a global threat to public health (3). Currently, due to the lack of effective treatment and vaccines, the best method to deal with this disease is to avoid infection and prevent its spread through protective behavior and personal health (4). Preventive behaviors are behaviors that prevent disease (5). Behaviors play an important role in providing and promoting health (6). Behavioral changes can be effective in stopping the spread of the disease (7). To stop the spread of infectious disease, prevention guidelines must be followed correctly by individuals (8). Simple precautions are effective in preventing the possibility of infection or the spread of COVID-19:

Wash your hands regularly with soap and water or disinfect your hands with an alcohol solution
Observe a distance of at least 1–2 m with other people (social distance)
Lack of touch of eyes, nose, and mouth Respiratory hygiene during sneezing and coughing.

Stay at home and refrain from attending rallies (9, 10). Many of these behaviors interfere with daily activities, so health advice should be followed voluntarily by individuals and they should adhere to lifestyle changes (8). The results of a study in Greece showed that most of the behaviors that people observed included not contacting people at high risk of the disease and patients with respiratory symptoms. Minimal measures included daily body temperature monitoring, monitoring of cough or shortness of breath, and use of masks in public places (11). Studying individual health-related behaviors and accepting the role of individual behavior in creating and maintaining health is effective in promoting health and preventing disease and evaluating health behaviors (6). Preventing the outbreak of COVID-19 disease is one of the important goals of the health system. It is necessary to study the status of preventive behaviors in developing intervention programs. This study aimed to determine the behaviors preventing the outbreak of COVID-19 to use the results of the study in designing appropriate interventions.

## Materials and Methods

### Study Design and Sample

This is a part of the results of a descriptive cross-sectional study conducted from April 9, 2020 to May 12, 2020 to investigate the behaviors preventing the outbreak of COVID-19 on 1,200 Iranians who have access to social networks (Telegram and WhatsApp) in different cities of Iran. Data were collected online, via a self-reported questionnaire, to calculate the sample size, the formula for estimating the sample size for the mean was used. Ninety-five percentage confidence interval and standard deviation were considered 0.66 according to the 5-choice questions. The accuracy of the estimate was considered 0.04. The minimum required sample was estimated at 1,200 participants. Inclusion criteria included people living in Iran who had access to the questionnaire on social networks and exclusion criteria included people who had a history of hospitalization due to COVID-19. Considering that at the beginning of the questionnaire, the purpose of the study was explained and it was stated that there is no need to write a name and the information will be confidential. After this explanation, those who wished to participate in the study completed the questionnaire.

### Measurement Tool and Data Analysis

The self-reported questionnaire was developed by the authors. The questionnaire includes two sections of demographic information (gender, marital status, education, and employment, place of residence, economic status, and history of COVID-19 among friends) and 19 questions related to preventive behaviors of COVID- 19. The output Excel file was transferred to the software SPSS, All questionnaires were evaluated in terms of data quality and outlier data Questionnaires with 10% percent (and more) of uncompleted questions, were excluded from the analysis. Data analysis was performed using SPSS software version 21 based on descriptive and analytical statistical tests *t*-test and analysis of variance at a significant level <0.05.

### Independent Variables

For sociodemographic variables, gender was coded as one for men, and tow for women. Education was categorized into Elementary, middle school, diploma, and university. Work status was broken down into government employee (reference category), non-government employee, retiree, self-employed, and unemployed, Housewives, and health workers. Evaluation from the economic level was divided into 5 categories (very good, good, average, poor, and very poor. In terms of marriage, participants were divided into three categories: single, married and divorced, or a deceased spouse. Also, according to the history of friends being infected with COVID-19 people were divided into three categories (Yes, No. Lack of information).

### Dependent Variables

Nineteen questions related to preventive behaviors of COVID- 19 with a 5-point criterion based on options (never, Very little, sometimes, most of the time, always). The range of scores of the questionnaire is 19–95. Validity index (CVI) and content validity ratio (CVR) were calculated 1 and the reliability of a questionnaire was 0.89.

### Analysis Methods

Data were analyzed based on descriptive and analytical statistics (*t*-test and one-way analysis of variance) at a significant level of <0.05.

### Ethical Approval

All the procedures in this study were approved by the Research Review Board (Research Code: 5329) with the Ethics of committee of Birjand University of Medical Sciences (IR.bums.REC.1399.003).

In order to observe the principles of ethics in research, in addition to the voluntary participation of individuals in the study, the purpose of the research was explained to participants, and questionnaires were collected and analyzed without mentioning the name.

## Results

### Social and Demographic Characteristics

The average age of the samples was 37.77 ± 11.20 years, 70.6% females and 76.5% were married. Table 1 shows demographic characteristics of the participants in detail.

**Table 1:** Frequency distribution of demographic variables of participants.

**Variables**		**Number**	**Percent**
Gender	Male	362	30.2
	Female	836	69.8
Education	Primary school	15	1.3
	Secondary school	24	2
	Diploma	172	14.4
	Academic	987	82.4
Occupation	Housewife	211	18.5
	Student	134	11.8
	Self-employed	172	8.1
	Worker	23	2
	Employed	420	36.9
	Retired	92	7.3
	Health worker	154	13.5
Marital status	Married	917	76.5
	Single	256	21.4
	Divorced/deceased spouse	25	2.1
History of infection among friends	Yes	196	16.4
	No	889	74.2
	Lack of information	113	9.4
Economic status	Very good	45	3.8
	Good	366	30.6
	Moderate	629	52.5
	Weak	158	13.2
Health condition	Very good	214	17.9
	Good	700	58.4
	Moderate	274	22.9

Among the questions that measure people's performance in preventing behaviors from COVID-19, the question related to not using hookah with 83% had the highest frequency and the question related to exercising and physical activity at home with 16% had the lowest frequency (Table 2).

**Table 2:** Frequency of subjects' answers to questions related to preventive behaviors from COVID-19.

**Behavior**	**Never(%) *N***	**Very little (%) *N***	**Sometimes (%) *N***	**Most of the time (%) *N***	**Always (%) *N***
Wash your hands regularly with soap and water	(2.25) 27	(2) 24	(5.3) 64	(40) 486	(49) 598
Refrain from kissing and shaking hands with others	(4.50) 54	(1.16) 14	(1.5) 18	(14.34) 172	(78) 941
Use your handkerchief or elbow when coughing and sneezing	(0.8) 10	(1.41) 17	(4.17) 50	(24.1) 290	(69.39) 832
Keep a distance of 1-2 meters from others	(0.7) 9	(3.08) 37	(12.92) 155	(37.69) 452	(45.53) 546
Don't go to crowded places and stay at home as much as possible	(1.16) 14	(2.91) 35	(10.17) 122	(38.28) 459	(47.45) 569
Daily disinfection of surfaces at home or at work that come in contact with hands	(1.16) 14	(6) 72	(18.01) 216	(34. 69) 416	(40.11) 481
Dispose of handkerchiefs, gloves and masks used healthy and safely	(1.25) 15	(4.92) 59	(9.92) 119	(27.60) 331	(56.29) 675
Wearing a mask when confronted with a person suspected of having coronavirus disease or being in crowded places	(1) 12	(2.50) 30	(5.50) 66	(25.27) 303	(65.72) 788
Avoid contact with hands, nose, mouth and eyes	(0.75) 9	(3.33) 40	(10.58) 126	(35.86) 430	(49.54) 594
Wear gloves when in contact with contaminated objects and surfaces	(2.41) 29	(6.92) 83	(12.26) 147	(29.44) 353	(48.95) 587
Exercise and physical activity at home	(10) 120	(28.52) 342	(29.85) 358	(15.42) 185	(16.18) 194
Eating healthy foods	(0.58) 7	(2.58) 31	(11.09) 133	(41.53) 498	(44.20) 530
Eat at least 3 servings of fruits and vegetables a day	(3.41) 41	(15.17) 182	(34.69) 416	(25.02) 300	(21.68) 260
Proper ventilation of the home or work environment by intermittent window opening	(1.08) 13	(5.42) 65	(17.59) 211	(34.69) 416	(41.20) 494
Prevent unnecessary attendance at medical centers	(2.83) 34	(3) 36	(5.08) 61	(24.43) 293	(64.63) 775
Disinfect personal items	(0.83) 10	(2.75) 33	(12.59) 151	(31.35) 376	(52.46) 629
No smoking	(11.42) 137	(1.58) 19	(1.16) 14	(3.75) 45	(82.06) 984
No hookah use	(10.42) 125	(1.16) 14	(1.25) 15	(3.58) 43	(83.56) 1,002
Adherence to the principles of social distance and non-travel	(2) 24	(1.83) 22	(4.67) 56	(25.52) 306	(65.97) 791

Forty-nine percentage of participants wash their hands regularly with soap and water. Seventy-eight percentage of people avoid kissing and shaking hands. 45.53% of people observed social distance. 65.72% of people have used masks in the face of suspicious people or crowded places. 65.97% of people adhere to the principles of social distance and have not traveled.

The mean score of COVID-19 preventive behaviors in the participants was 62.67 ± 8.53. There was a significant relationship between the variable gender, education, economic status and preventive behaviors of COVID-19 (Table 3).

**Table 3:** The mean score of preventive behaviors based on demographic variables.

**Variables**		**Mean ± SD**	***P*-Value**
Gender	Male	60.21 ± 9.13	<0.001
	Female	63.74 ± 8.03	
Education	Primary School	60.13 ± 12.77	<0.001
	Secondary School	56.59 ± 12.9	
	Diploma	60.43 ± 9.27	
	Academic	63.89 ± 8.00	
Occupation	Housewife	8.55 ± 62.63	0.02
	Student	62.09 ± 9.31	
	Self-employed	8.00 ± 61.08	
	Worker	58.43 ± 7.45	
	Employed	8.55 ± 63.08	
	Retired	64.08 ± 8.06	
	Health worker	63.12 ± 7.53	
Marital status	Married	62.79 ± 8.50	0.02
	Single	61.87 ± 8.69	
	Divorced/deceased spouse	66.60 ± 6.57	
History of infection among friends	Yes	62.60 ± 7.31	0.003
	No	63.02 ± 8.64	
	Lack of information	60.10 ± 9.20	
Economic status	Very good	65.68 ± 7.87	0.001
	Good	64.21 ± 7.83	
	Moderate	8.44 ± 62.36	
	Weak	9.55 ± 59.51	

## Discussion

Considering the role of preventive behaviors in reducing the prevalence of COVID-19, the main way to contain the spread of the virus is to support changes in individuals' behaviors and their compliance with health prescriptions (12). This study was designed and conducted to investigate the preventive behaviors of COVID-19 and its relationship with some demographic variables. The range of achievable scores for preventive behaviors of COVID-19 were (19–95) and the mean score of preventive behaviors is 62.67 ± 8.53. The results of the study showed that the mean score of preventive behaviors in women was higher than men and more than the overall mean score of preventive behaviors in both sexes and there was a statistically significant difference between the mean scores of preventive behaviors in both sexes. In a study conducted by Choi et al., The results showed that preventive behaviors were higher in women, which is consistent with the present study (5). The results of other studies also showed that women performed better in preventive behaviors (13, 14). The highest average score of preventive behaviors is related to people who have a university education level. The results of a study showed that the use of masks has a significant relationship with the level of education (15). In one study, the level of the correct response to preventive behaviors in relation to MERS was 0.22 and Preventive behaviors have a significant relationship with education level (16). In the present study, there is a significant relationship between people's jobs and preventive behaviors. Occupational conditions play an important role in preventive behaviors. The results of the study showed that there is a significant relationship between economic level and preventive behaviors. The average score of preventive behaviors was the lowest in people who assessed their economic situation as weak. Many behaviors, such as wearing masks, gloves, eating healthy foods, disinfecting surfaces, and washing hands regularly, require people to spend money. It is necessary for governments to intervene to improve the economic situation of people with low economic status. Advertising and public health promotion activities supported by government agencies provide cues to increase the use of face masks to prevent respiratory infection (15). In one study, self-report of infection prevention behaviors was not desirable and people especially needed training in the use of personal protective equipment (17). Preventive behavior affected the most significantly by attitude and risk perception (5). Health behaviors are not necessarily interdependent, and individuals can only perform a number of health behaviors. Each behavior pursues a specific goal based on individual experience. It is important that persons recognize that preventive behavior can prevent infectious and could stop its spread (5). In the present study, the frequency of preventive behaviors is different, so that the most behavior is related to not using hookah and smoking and the least behavior is related to regular physical activity at home. In one study, 65.5% of people were able to avoid being in the community (18). In one study, among the flu-preventing behaviors, washing hands with soap and water, covering the mouth and nose when coughing and sneezing were the most common. The use of masks has had the least frequency of behavior (19). The results of another study showed that during the corona epidemic, 97.4% of people tried not to leave the house, 93.6% wore masks when leaving the house, and 91.5% did not go to crowded and closed places (20). In another study, 83.4% of people avoided crowded places and washed their hands regularly, and only 51.2% of people wore masks (21). In a study in Wuhan, China, on preventive behaviors at the time of the outbreak of Quaid 19, 8.5% of those surveyed used public transportation at the time of the outbreak, 2.4% were in crowded places, and 95.2% of They used masks, 100% of people washed their hands regularly with soap and water, and 73.8% disinfected living areas (22). In the study, 40% of respondents most often wore masks in public places and washed their hands regularly (10 times a day), and about one-third of people refused to visit crowded places in China (15). In another study, regular hand washing was moderate, with less than half of people wearing surgical masks (17). In a study in China, 98% of people wore masks when leaving home (14). Regarding their quarantine behavior, only 7% of people were interested in quarantine (23). In one study, the average rate of preventive behaviors in medical students was 47.14 (24). Due to some differences that were observed in the results of the present study with some other studies, it should be noted that the behavior of individuals depends on many factors, including circumstances and situations. Differences in the psychological, cultural, social, and demographic characteristics of the participants, as well as differences in tools, may be reasons for the discrepancies in the studies.

### Study Strengths and Limitations

The results may help health authorities to plan preventive strategies. One of the advantages of the study is that many people from different parts of Iran have entered the study. Due to COVID- 19 disease, this method of data collection was appropriate. One of the limitations of this study is completing the questionnaires only through cyber space. People included in the study who had access to the Internet and social networks. Therefore, in order to generalize to the whole community, it should be interpreted with more caution.

Also, because of the people in cities have more access to social media than villagers, and women use social networks more than men and have more time to complete the questionnaire. Therefore, the majority of study participants were women and urban dwellers. Also data used in the analysis of this study were self-reported, which might suffer from reporting bias.

## Conclusions

Behaviors play an important role in providing and promoting health. To stop the spread of infectious disease, prevention guidelines must be followed correctly by individuals. The results showed that there was a significant relationship between the variables of gender, education, economic status, and preventive behaviors of COVID- 19. It is important to design educational and supportive interventions to improve the level of physical activity and perform coronavirus prevention behaviors with more focus on men, illiterate people, and poor economic status.

## Data Availability Statement

The data analyzed in this study is subject to the following licenses/restrictions: The datasets generated and/or analyzed during the current study are not publicly available due to privacy and confidentiality agreements as well as other restrictions but are available from the corresponding author on reasonable request. Requests to access these datasets should be directed to Mitra Moodi, mitra_m2561@yahoo.com.

## Ethics Statement

The studies involving human participants were reviewed and approved by This research is the result of a research project approved by Birjand University of Medical Sciences with number 5,329 and Ethics Code IR.BUMS.REC. 1399.003. Written informed consent from the participants' legal guardian/next of kin was not required to participate in this study in accordance with the national legislation and the institutional requirements.

## Author Contributions

All authors made substantial contributions to conception and design, acquisition of data, or analysis and interpretation of data, took part in drafting the article or revising it critically for important intellectual content, gave final approval of the version to be published, and agreed to be accountable for all aspects of the work.

## Conflict of Interest

The authors declare that the research was conducted in the absence of any commercial or financial relationships that could be constructed as a potential conflict of interest.
